# FAAH inhibition enhances anandamide mediated anti-tumorigenic effects in non-small cell lung cancer by downregulating the EGF/EGFR pathway

**DOI:** 10.18632/oncotarget.1723

**Published:** 2014-02-21

**Authors:** Janani Ravi, Amita Sneh, Konstantin Shilo, Mohd W. Nasser, Ramesh K. Ganju

**Affiliations:** ^1^ Department of Pathology, The Ohio State University, Ohio, USA

**Keywords:** NSCLC, EGFR, Met-F-AEA, FAAH

## Abstract

The endocannabinoid anandamide (AEA), a neurotransmitter was shown to have anti-cancer effects. Fatty acid amide hydrolase (FAAH) metabolizes AEA and decreases its anti-tumorigenic activity. In this study, we have analyzed the role of FAAH inhibition in non-small cell lung cancer (NSCLC). We have shown that FAAH and CB1 receptor which is activated by AEA are expressed in lung adenocarcinoma patient samples and NSCLC cell lines A549 and H460. Since the synthetic analogue of anandamide (Met-F-AEA) did not possess significant anti-tumorigenic effects, we used Met-F-AEA in combination with FAAH inhibitor URB597 which significantly reduced EGF (epidermal growth factor)-induced proliferative and chemotactic activities in vitro when compared to anti-tumorigenic activity of Met-F-AEA alone. Further analysis of signaling mechanisms revealed that Met-F-AEA in combination with URB597 inhibits activation of EGFR and its downstream signaling ERK, AKT and NF-kB. In addition, it inhibited MMP2 secretion and stress fiber formation. We have also shown that the Met-F-AEA in combination with URB597 induces G0/G1 cell cycle arrest by downregulating cyclin D1 and CDK4 expressions, ultimately leading to apoptosis via activation of caspase-9 and PARP. Furthermore, the combination treatment inhibited tumor growth in a xenograft nude mouse model system. Tumors derived from Met-F-AEA and URB597 combination treated mice showed reduced EGFR, AKT and ERK activation and MMP2/MMP9 expressions when compared to Met-F-AEA or URB597 alone. Taken together, these data suggest in EGFR overexpressing NSCLC that the combination of Met-F-AEA with FAAH inhibitor resulted in superior therapeutic response compared to individual compound activity alone.

## INTRODUCTION

Non-small cell lung cancer (NSCLC) is a metastatic form of cancer which accounts for about 85% of lung cancer cases and is the primary cause of cancer related deaths in the United States. Though tobacco smoking is the major cause of lung cancer, about 15-20% of cases are attributed to non-smokers and involve the activation of various signaling pathways for tumor development [1]. Adenocarcinoma and squamous cell carcinoma, the two most common histological subtypes of lung cancer are categorized as NSCLC. Poor prognosis and chemotherapeutic resistance which may be due to modulation of key cell signaling mechanisms pose major concerns [2-3].

The cannabinoid family is categorized into endogenous cannabinoids (produced inside the body), phytocannabinoids (plant derived) and synthetic cannabinoids which activate the specific G-protein coupled receptors CB1 and CB2. CB1 receptor is mainly expressed in the brain and CNS whereas CB2 receptor is expressed in immune system [4-6]. The use of cannabinoid agonists as anti-cancer agents has proven successful in various *in vitro* and *in vivo* cancer models such as glioma, breast, prostate, colon, leukemia and lymphoid tumors [7-10]. They have been shown to modulate various cell survival pathways such as the extracellular signal-related kinase (ERK), phosphoinositide 3-kinase (PI3K), p38 mitogen-activated protein kinase (p38 MAPK), protein kinase B (AKT) and ceramide pathways [11-13]. Anandamide (AEA) and 2-arachidonoylglycerol (2-AG) are the two well characterized endocannabinoids which are endogenous ligands for the cannabinoid receptors. Although endocannabinoids were initially studied for their neurological and psychiatric effects, there is increasing evidence of their contribution to inflammation and tumorigenesis [14-15]. AEA, which is mainly synthesized from phospholipids, is inactivated by enzyme fatty acid amide hydrolase (FAAH) mediated hydrolysis to arachidonic acid (AA) and ethanolamine (EA), whereas 2-AG is hydrolyzed into AA and glycerol [16-20]. Thus, the effects of the endocannabinoids are profoundly affected by their enzyme mediated hydrolysis. Moreover, inactivation of FAAH activity has been shown to potentiate the anti-tumorigenic effects of AEA in prostate cancer [21]. However, the exact roles of FAAH and its regulation of AEA activity have not been elucidated in the context of tumorigenicity in NSCLC. In our work, we focus on AEA, an endogenous cannabinoid agonist specific for the CB1 receptor and the effect of FAAH inhibition on the activity of AEA.

The genetic abnormalities associated with lung cancer are attributed to alterations in the signaling pathways which are targets for drug therapies. Most of these stimulatory signaling pathways are driven to a malignant phenotype characterized by uncontrolled proliferation and an apoptosis escape mechanism. Epidermal growth factor receptor (EGFR) is a family of four Receptor tyrosine kinases (RTKs) EGFR (ERBB1, HER1), ERBB2 (HER2, Neu), ERBB3 (HER3) and ERBB4 (HER4) [22-23]. EGFR dysregulation is associated with multiple cancer types including malignant transformations and metastasis [24]. EGFR overexpression and signaling pathway gene mutations play a vital role in lung tumorigenesis. Recent evidence suggests that cancer cells undergo escape mechanisms to defend against the host system by activation of alternative growth signaling pathways [25]. The cell cycle in eukaryotes is regulated by a family of cyclins and cyclin dependent kinases (CDKs), which are members of protein kinase complexes. Each complex consists of a cyclin (regulatory subunit) which binds to a CDK (catalytic subunit) to form an active cyclin-CDK complex that gets activated at various checkpoints during the cell division cycle [26-27]. Several studies indicate that cell cycle markers are mutated in most malignant cancers and might lead to Programmed Cell Death (PCD), where cells undergo suicide program [26-28]. Apoptosis is a type of PCD which involves the activation of caspases and DNA fragmentation [29-31]. Cell cycle dysregulation and resistance to apoptosis are often attributed to abnormal EGFR signaling [22, 32]. Hence, identification of novel receptors expressed in tumor cells that target against EGFR activation will be a promising strategy against NSCLC.

In our present study, we analyzed the effect of AEA on lung tumorigenesis when FAAH is inhibited. We show that Met-F-AEA in combination with URB597 reduces NSCLC growth *in vitro* and *in vivo*. Our results reveal that the combination treatment inhibits the activation of EGFR and its downstream signaling targets. These findings suggest the possibility of exploring the components of endocannabinoid system as a novel therapeutic target for NSCLC treatment.

## RESULTS

### Primary lung cancer tissues and NSCLC cell lines express CB1 and FAAH

**Figure 1:**
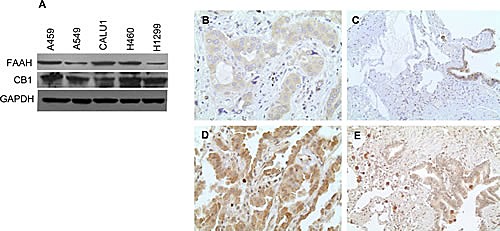
NSCLC cell lines and primary lung cancer tissues express CB1 and FAAH (A) NSCLC cell lines were subjected to immunoblot analysis to determine the expression of CB1 and FAAH. GAPDH served as loading control. Representative photomicrographs of IHC staining of FAAH expression in lung adenocarcinoma (B) and respiratory epithelia (C). Representative photomicrographs of IHC staining of CB1 expression in lung adenocarcinoma (D) and respiratory epithelia (E).

Anandamide is known to mediate its effects through cannabinoid receptor CB1 [7, 30]. Hence, the expression of FAAH and CB1 were assessed in the NSCLC cell lines- A459, A549, CALU1, H460 and H1299 (Fig 1A). All the cell lines expressed FAAH and CB1 as detected by Western Blot. Also, we utilized tissue microarray (TMA) to detect levels of FAAH and CB1 in cancer patients. Eleven of fifty seven (19.3%) lung adenocarcinomas showed high cytoplasmic expression of FAAH (Fig 1B, C). Twenty two of thirty five (62.9%) lung adenocarcinomas showed high cytoplasmic expression of CB1 (Fig 1D, E).

### FAAH inhibition enhances the anti-proliferative activity of Met-F-AEA in NSCLC cell lines

**Figure 2:**
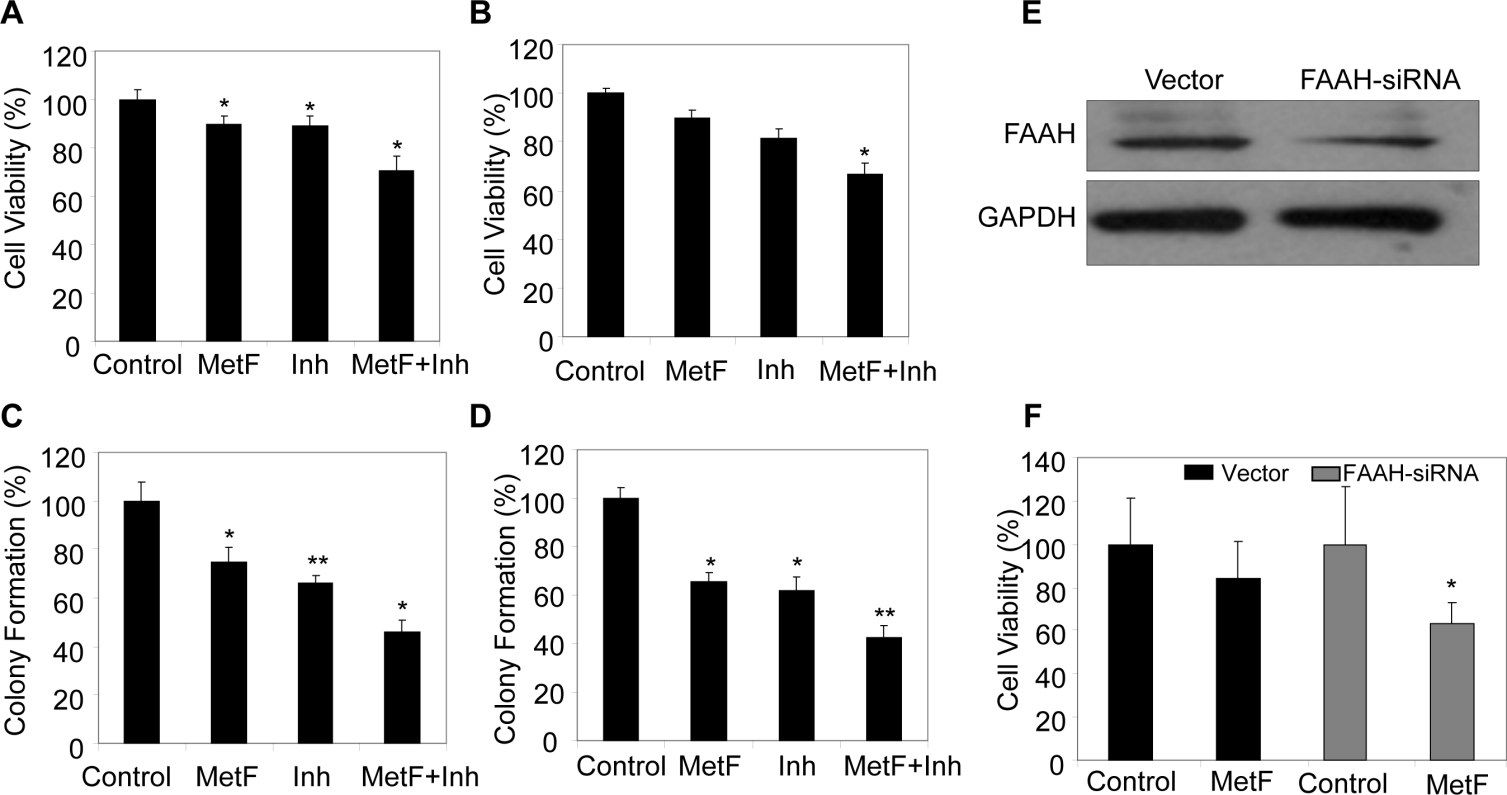
FAAH inhibition enhances the anti-proliferative activity of Met-F-AEA in NSCLC cell lines A549 (A) and H460 (B) cells were serum starved for 24h and treated with control, Met-F-AEA (MetF, 10μM), FAAH inhibitor URB597(Inh, 0.2μM) or MetF+Inh and analyzed for viability by MTT assay. 1000 individual A549 (C) and H460 (D) cells were plated in six well plates and subjected to colony formation assay by treating with control, Met-F-AEA (MetF, 10μM), FAAH inhibitor URB597 (Inh, 0.2μM) or MetF+Inh as shown for six days. The colonies were then fixed, stained and counted. (E) H460 cells were transfected with 100pmol of either the non targeting control (vector) or FAAH-siRNA (Dharmacon) using Lipofectamine 2000 (Invitrogen) according to the manufacturer's instructions for 36h and the expression of FAAH in siRNA and non targeted cells were evaluated by Western blot. (F) H460 cells which were transfected with either FAAH siRNA or non targeted control (vector) for 36h were treated with Met-F-AEA for 24h and subjected to MTT assay. P<0.05 (*) and P<0.005 (**) as calculated by Student's t test. Data represent the mean ± SD per experimental group.

Proliferation is one of the characteristic features of cancer cells to grow and multiply [33-35]. To analyze whether inhibiting FAAH can enhance the activity of Met-F-AEA (synthetic analogue of anandamide), we evaluated the potential of Met-F-AEA in combination with FAAH inhibitor- URB597 as a possible therapeutic target. We treated the NSCLC cell lines- A549 and H460 with either FAAH inhibitor- URB597 or Met-F-AEA or in combination and observed the effects after 24h. MTT assay showed a significant decrease in cell viability in the combination treatment when compared to Met-F-AEA alone (Fig 2A, B). Also, we performed clonogenic assay which measures the ability of single cell to form clones. Combination treatment significantly reduced the number of colonies when compared to Met-F-AEA or URB597 alone in both the cell lines (Fig 2C, D).

To enhance the effect of the endocannabinoid Met-F-AEA, we used the siRNA approach to knockdown FAAH in H460 cells. To analyze the efficiency of knockdown, the transfected cells were subjected to Western Blot analysis, which showed reduced expression of FAAH (Fig 2E). Cells transfected with FAAH siRNA were more sensitive to Met-F-AEA treatment than the control siRNA transfected cells (Fig 2F). These data suggest that Met-F-AEA in combination with URB597 inhibits cell growth *in vitro*.

### FAAH inhibition enhances the anti-migratory and anti-invasive activities of Met-F-AEA in NSCLC cell lines

**Figure 3:**
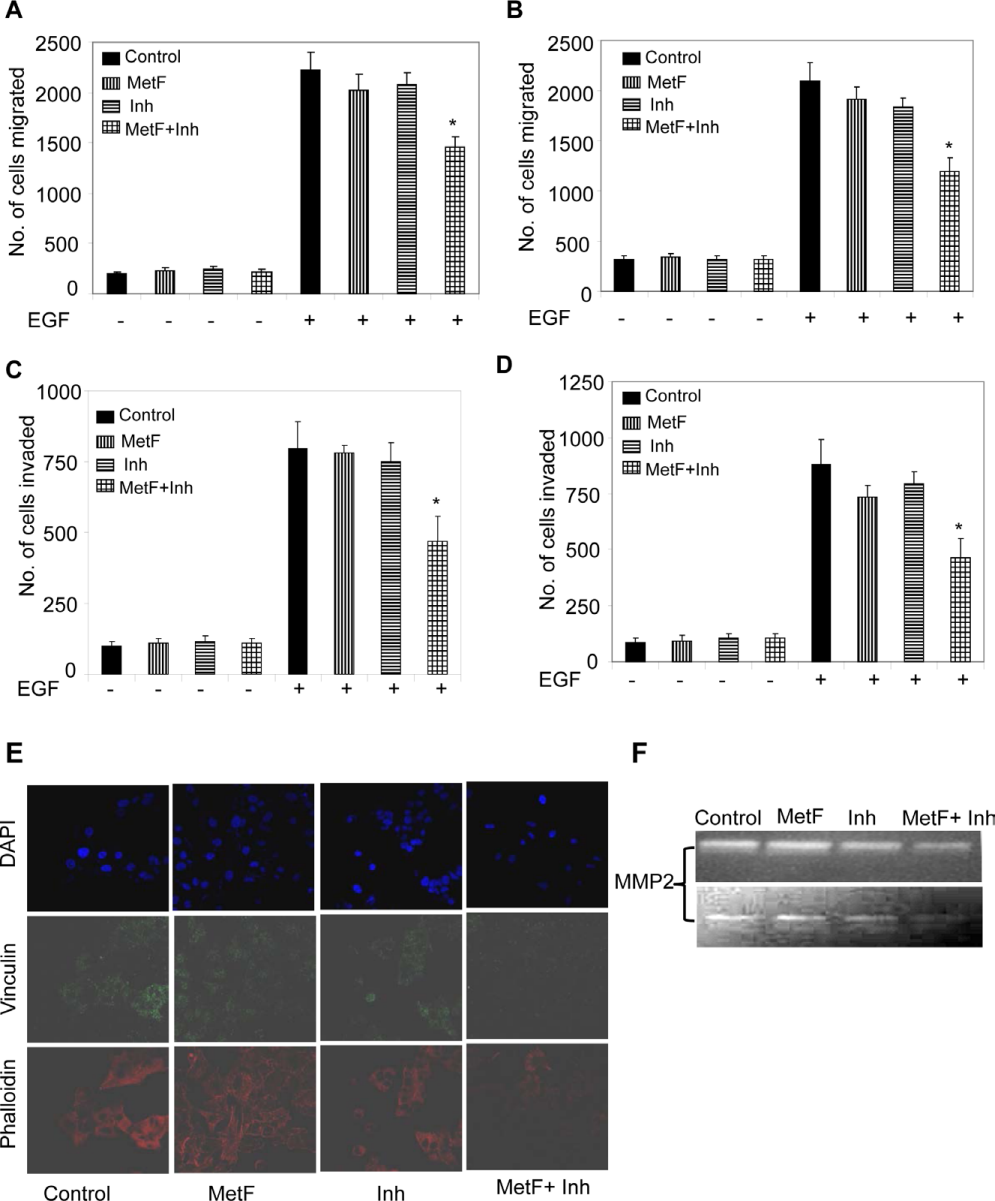
FAAH inhibition enhances the anti-migratory and anti-invasive activities of Met-F-AEA in NSCLC cell lines A549 (A) and H460 (B) cells were treated with control, Met-F-AEA (MetF, 10μM), FAAH inhibitor URB597 (Inh, 0.2μM) or MetF+Inh for 24h and subjected to EGF (100ng/ml)-induced migration using transwell plates. The number of cells migrated were stained with Hema stain and counted in five different fields. A549 (C) and H460 (D) cells were treated with control, Met-F-AEA (MetF, 10μM), FAAH inhibitor URB597 (Inh, 0.2μM) or MetF+Inh for 24h and subjected to EGF (100ng/ml)-induced invasion assay using transwell plates coated with matrigel. Invaded cells were stained with Hema stain and counted. (E) Confocal microscopy visualization of A549 cells treated with control, Met-F-AEA (MetF, 10μM), FAAH inhibitor URB597 (Inh, 0.2μM) or MetF+Inh for 24h and stimulated with EGF (100ng/ml) and stained for phalloidin (red), vinculin (green) expression and DAPI (blue). (F) A549 (upper panel) and H460 (lower panel) cells were treated with control, Met-F-AEA (MetF, 10μM), FAAH inhibitor URB597(Inh, 0.2μM) or MetF+Inh for 48h and the supernatants were collected, concentrated and run on zymogram gels to detect the active form of MMP2. P<0.05 (*) as calculated by Student's t test. Graphs represent the mean ± SD for each experiment repeated three times with similar results.

Migration and invasion are required for cancer cells to spread within the specific tissue and form the characteristic features of angiogenesis and metastasis [36]. The extracellular matrix (ECM) acts as a barrier towards this cell motility process. Chemoattractants like EGF provide a directed migration for cancer cells [22, 32, 36]. We further analyzed the effects of Met-F-AEA in combination with URB597 on EGF induced chemotaxis and observed significant inhibition in EGF induced cell motility (S1), migration (Fig 3A, B) and invasion (Fig 3C, D) when compared to Met-F-AEA or URB597 alone in A549 and H460 cells.

To migrate through the ECM, cancer cells possess extended protrusions that are rich in actin filaments and adhesion molecules [37]. We investigated the effects of Met-F-AEA when FAAH is inhibited on EGF induced actin stress fiber and focal adhesion formation, which were detected by changes in actin and vinculin expressions, respectively. We observed that cells treated with the combination treatment significantly inhibited EGF induced actin and vinculin expressions more effectively than Met-F-AEA or URB597 alone. Further examination with the confocal microscopy revealed the decreased presence of migratory structures such as lamellipodia in the cells treated with the combination treatment when compared to control (Fig 3E).

Tumor invasion begins with protrusion of the ECM and basement membrane. The matrix metalloproteinase (MMP) family has been shown to be responsible for degradation of the ECM, which is one of the initial steps in metastasis. Higher expressions of MMP2 and MMP9 have also been correlated with poor prognosis in early stages of lung adenocarcinoma [38]. In our present study, we observed a significant decrease in secretion of MMP2 by zymogram when cells were treated with combination treatment, Met-F-AEA along with URB597 (Fig 3F). These data suggest that the Met-F-AEA in combination with URB597 inhibits cell migration and invasion in NSCLC cell lines.

### FAAH inhibition enhances Met-F-AEA mediated inhibition of EGFR signaling in NSCLC cell lines

**Figure 4:**
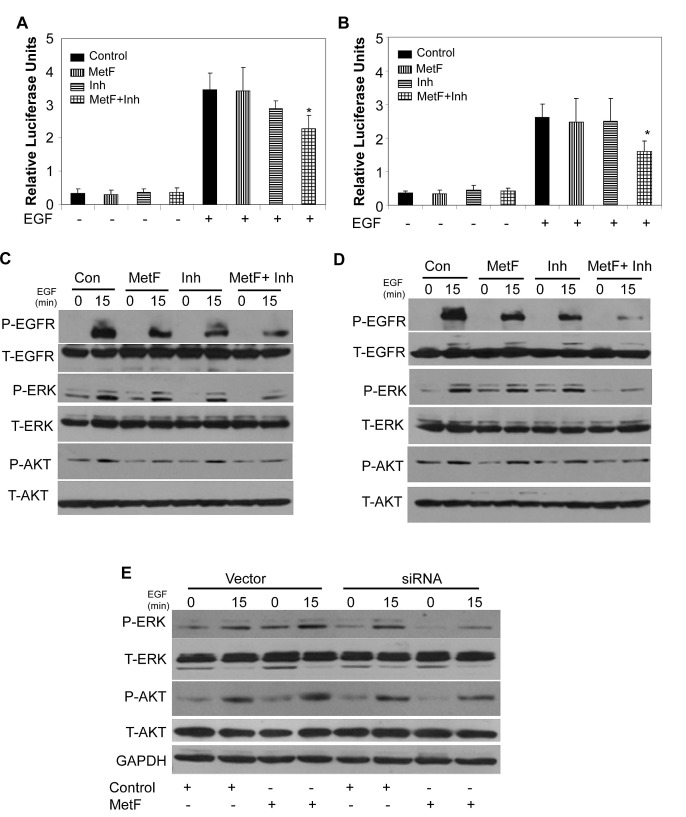
FAAH inhibition enhances Met-F-AEA mediated inhibition of EGFR signaling in NSCLC cell lines A549 (A) and H460 (B) cells were treated with control, Met-F-AEA (MetF, 10μM), FAAH inhibitor URB597(Inh, 0.2μM) or MetF+Inh for 24h and transfected with either wild-type or NF-kB plasmid for 24h, stimulated with EGF (100ng/ml) for additional 24h, lysed and analyzed for luciferase activity. Renilla luciferase vector served as internal control. A549 (C) and H460 (D) cells were treated with control, Met-F-AEA (MetF, 10μM), FAAH inhibitor URB597(Inh, 0.2μM) or MetF+Inh for 24h, stimulated with EGF (100ng/ml) for 0 and 15 min and subjected to Western blot analysis to determine expression of phopho-EGFR, ERK or AKT (P-EGFR, P-ERK, P-AKT) and total EGFR, ERK and AKT (T-EGFR, T-ERK, T-AKT). (E) H460 cells which were transfected with either FAAH siRNA or non targeted control (vector) for 36h were treated with either Met-F-AEA or control for 24h, stimulated with EGF (100ng/ml) for 0 and 15 min and analyzed by Western blotting to determine expression levels of phopho-ERK or AKT (P-ERK, P-AKT) and total ERK and AKT (T-ERK, T-AKT). P<0.05 (*) as calculated by Student's t test. Data represent the mean ± SD for each experiment repeated three times with similar results.

NF-kB is involved in invasion and metastasis in various cancer types [39]. Previous studies have shown that NF-kB activation is involved in EGFR mediated lung tumor progression [39-40]. Therefore, we sought out to determine if FAAH inhibition can enhance Met-F-AEA mediated inhibition of EGF induced NF-kB activation by luciferase reporter assay. Cells were transfected with either vector or NF-kB plasmid and the translocation of NF-kB into the nucleus was studied in the presence or absence of EGF. Met-F-AEA in combination with URB597 treated cells underwent significantly reduced EGF induced NF-kB translocation into the nucleus when compared to Met-F-AEA or URB597 alone (Fig 4A, B).

The EGFR signaling pathway activates diverse cellular targets which are crucial for cell proliferation, survival, angiogenesis, migration and adhesion and are often dysregulated in cancer cells. AKT and ERK are important survival molecules that are essential for EGF induced cell growth and motility [22, 25, 32]. To understand the molecular mechanism by which FAAH inhibition enhances Met-F-AEA and regulates EGFR pathway, we treated the cells with Met-F-AEA together with URB597, induced with EGF and observed reduction in the tyrosine phosphorylation of EGFR, serine phosphorylation of AKT and tyrosine phosphorylation of ERK which are the immediate downstream targets of EGFR pathway when compared to Met-F-AEA or URB597alone (Fig 4C, D).

To further validate that FAAH inhibition enhances the anti-tumorigenic effects of Met-F-AEA, H460 cells were transfected with FAAH siRNA, treated with Met-F-AEA or vehicle, stimulated with EGF and analyzed by Western blotting. Cells transfected with FAAH siRNA and treated with Met-F-AEA showed reduced expression levels of P-AKT and P-ERK when compared to Met-F-AEA alone (Fig 4E).

Taken together, these results indicate that Met-F-AEA in combination with URB597 downregulates EGFR receptor activation and also significantly inhibits the downstream signaling targets of EGFR pathway.

### FAAH inhibition enhances Met-F-AEA induced cell cycle arrest and apoptosis at later stage

**Figure 5:**
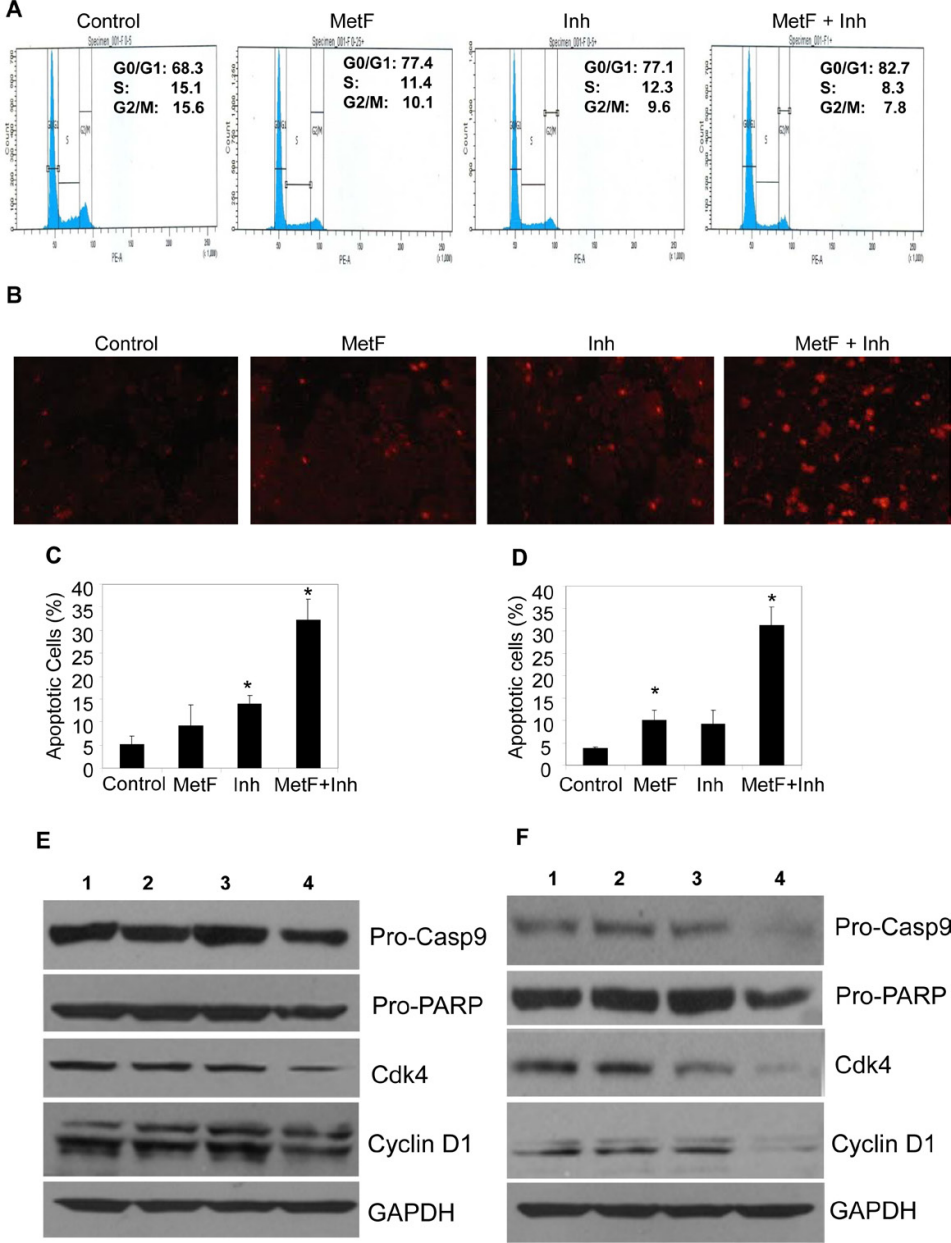
FAAH inhibition enhances Met-F-AEA induced cell cycle arrest and apoptosis at later stage (A) A549 cells were pre-treated with control, Met-F-AEA (MetF, 10μM), FAAH inhibitor URB597 (Inh, 0.2μM) or MetF+Inh for 48h, stained with propidium iodide and analyzed for cell cycle arrest by flow cytometry. (B) Representative images of TUNEL positive cells in H460 cells. H460 (C) and A549 (D) cells were pre-treated with control, Met-F-AEA (MetF, 10μM), FAAH inhibitor URB597 (Inh, 0.2μM) or MetF+Inh for 48h and subjected to TUNEL assay. The percentage of apoptotic cells was calculated as the percentage of fluorescent positive cells. A549 (E) and H460 (F) cells were pre-treated with control (1), Met-F-AEA (2), FAAH inhibitor URB597(3) or MetF+Inh (4) for 48h and subjected to Immunoblot analysis to determine the expression of cell cycle markers cyclin D1, CDK4 and apoptotic markers pro-caspase-9 and pro-PARP. GAPDH served as loading control. P<0.05 (*) as calculated by Student's t test. Data represent the mean ± SD for each experiment repeated three times with similar results.

Cell cycle dysregulation is frequently associated with cancer growth. EGFR activation leads to tumor cell survival, aberrant cell cycle and evasion of apoptosis, ultimately leading to resistance to cytotoxic therapies [22, 25, 32]. Hence, we sought to assess whether Met-F-AEA in combination with URB597 can induce cell cycle arrest leading to apoptosis. Cells were treated with control, Met-F-AEA, URB597 or combination treatment of Met-F-AEA with URB597 for 48h and subjected to cell cycle analysis. We observed significant cell cycle arrest in the G0/G1 phase in the combination therapy treated cells when compared to Met-F-AEA alone (Fig 5A). Met-F-AEA in combination with URB597 increased the percentage of apoptotic cells as shown by TUNEL assay (Fig 5B, C, D) and significantly inhibited pro-caspase9 and pro-PARP. Apoptosis was also induced by cell cycle arrest, as we observed significant decrease in cyclin D1 and CDK4, which are essential for G1/S phase progression (Fig 5E, F). These results show that Met-F-AEA together with URB597 induced apoptosis and is mediated by cell cycle blockade.

### FAAH inhibition enhances Met-F-AEA mediated inhibition of NSCLC tumor growth in vivo by downregulating EGFR signaling

**Figure 6:**
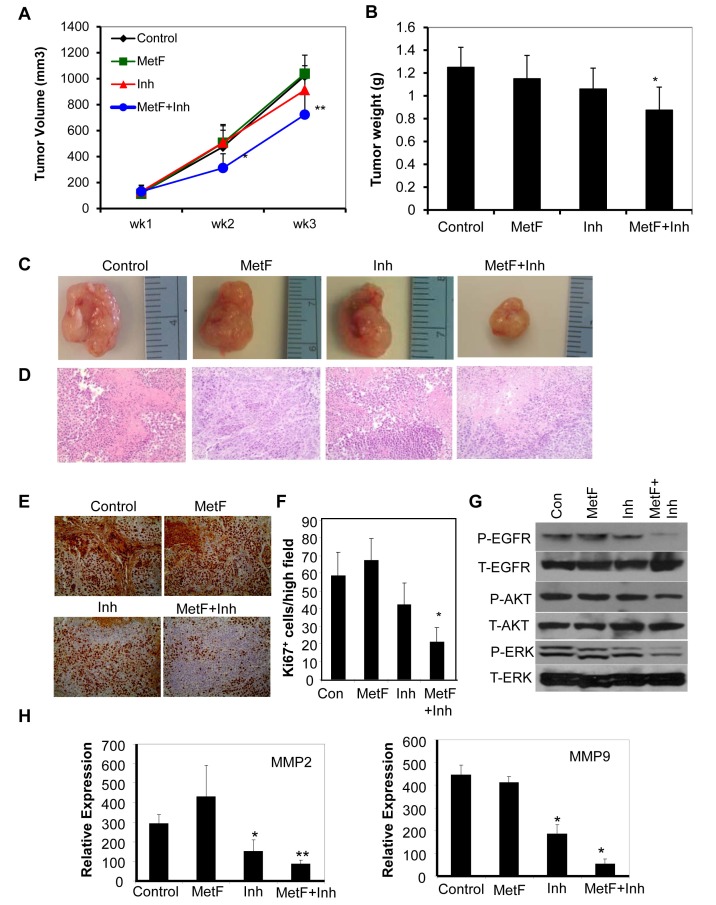
FAAH inhibition enhances Met-F-AEA mediated inhibition of NSCLC tumor growth *in vivo* by downregulating EGFR signaling (A) H460 cells were subcutaneously injected in nude mice and palpable tumors were treated with control, Met-F-AEA (MetF, 5mg/kg), FAAH inhibitor URB597 (Inh, 1mg/kg) or MetF+Inh every third day for 3 weeks. Tumor volume was assessed periodically and calculated using the formula= length × (width) ^2^ /2. (B) Tumor weight measured various experimental groups. (C) Representative tumors dissected from various experimental groups. (D) Representative photomicrographs of H&E and (E) Ki67 staining of tumors extracted from various experimental groups. (F) The number of Ki67 positive cells was counted in four different fields using bright field microscopy in each experimental group and the average was calculated. (G) Xenograft tumors isolated from (A) were subjected to Western blot analysis to determine expression of phospho-EGFR, ERK or AKT (P-EGFR, P-ERK, P-AKT) and total EGFR, ERK and AKT (T-EGFR, T-ERK, T-AKT). Control, Met-F-AEA (MetF), FAAH inhibitor URB597(Inh) or MetF+Inh. (H) Xenograft tumors were subjected to Real Time PCR analysis to determine the expression of MMP2 and MMP9. P<0.05 (*) and P<0.005 (**) as calculated by Student's t test. Data represent the mean ± SD for each experiment repeated three times with similar results.

To evaluate the tumor suppressive effects of Met-F-AEA in combination with URB597 *in vivo*, we determined the anti-tumorigenic potential of the combination treatment on H460 cells in nude mouse model. We induced tumors by injecting H460 cells subcutaneously into the right flank of male nude mice. When the tumors reached a palpable size, we treated them with ethanol control, Met-F-AEA, URB597 or Met-F-AEA in combination with URB597 every third day for three weeks. Tumor volume was monitored throughout the treatment period (Fig 6A). We observed a dramatic decrease in tumor formation in the combination regime treated mice as compared to control (Fig 6B, C). H&E staining revealed that the combination regime treated tumors were less aggressive and necrotic than the control tumors (Fig 6D). In addition, there was a significant decrease in the expression of proliferation marker Ki67 and hence, a reduced mitotic index in Met-F-AEA and URB597 combination treated tumors as compared to Met-F-AEA or URB597 treated tumors alone (Fig 6E, F).

To further determine the mechanism by which the tumors are inhibited, we isolated the tumor xenografts from the nude mice and extracted protein and RNA from them. The Met-F-AEA and URB597 combination treated tumors showed lesser phosphorylation of EGFR, ERK and AKT compared to Met-F-AEA or URB597 treated tumors alone (Fig 6G). Also, real time PCR analysis revealed that MMP2 and MMP9 levels were significantly downregulated in the URB597 treated tumors and Met-F-AEA in combination with URB597 treated tumors when compared to the control (Fig 6H), confirming with our *in vitro* findings.

## DISCUSSION

Lung cancer is estimated to be one of the most fatal cancer related malignancies in the world and fatality is often associated with drug resistance complications. Thus, there is an immediate requirement to develop innovative ways for the treatment of this disease [2-3]. Cannabinoids and their derivatives are being focused on cancer treatments because of their association with pain modulation, cell growth inhibition, anti-inflammation, tumor regression, cell cycle arrest and apoptosis. The endocannabinoid system is involved in a complex array of signaling pathways which might be receptor dependent or independent [7-10]. Though recent studies have shown that endocannabinoids exert potential anti-tumor effects in various cancer cells [41], not much is known about their effects in lung cancer. Here, we report, for the first time that Met-F-AEA and FAAH inhibitor URB597 significantly inhibited NSCLC growth. We have also explored the mechanisms by which Met-F-AEA in combination with URB597 inhibits NSCLC tumor growth.

In our present study, we found that FAAH and cannabinoid receptor CB1 are expressed in lung cancer patient samples as well as in NSCLC cell lines. Increased FAAH expression has been associated with poor patient survival in prostate and breast cancer [21, 42]. Also, overexpression of FAAH was found to be sufficient to increase migration and invasion in prostate cancer cells [21]. Our results show that Met-F-AEA that binds to CB1 does not have significant anti-tumorigenic effects *in vitro* and *in vivo*. This might be due to the conversion of AEA by enzyme FAAH into metabolites, leading to limitation in the concentration and action of the ligand. Previous reports show that AEA is converted into arachidonic acid (AA) and ethanolamine (EA) by FAAH enzyme [19-20]. AA is metabolized by COX2 and other enzymes to form prostaglandin (PGE_2_) and epoxyeicosatetraenoic acid (EE) [43-44]. These secondary metabolites have been shown to enhance tumor growth and metastasis in various cancer types [44-45]. Also, there are reports which suggest that methanandamide increases murine lung tumor growth by modulation of prostaglandin (PGE_2_) production and COX2 expression[44]. Therefore, in our study, we blocked FAAH pharmacologically (by using FAAH inhibitor URB597) and genetically (by siRNA approach) to enhance the anti-tumorigenic effects of Met-F-AEA. Our results show, for the first time, that Met-F-AEA, together with URB597 exerts anti-proliferative effects on NSCLC *in vitro* and *in vivo*.

In our study, we have shown that Met-F-AEA in combination with URB597 inhibits EGFR phosphorylation and downregulates EGFR mediated signal transduction pathways involving AKT and ERK, which are key cell survival molecules, *in vitro* and *in vivo*. This is important because EGF/EGFR axis is known to regulate cancer cells to proliferate and migrate to distant sites. Furthermore, aberrant EGFR expression and function lead to highly aggressive lung tumors, ultimately causing poor patient survival and increased resistance to conventional chemotherapeutic drugs [22, 24]. Thus, it would be interesting to identify novel targets that have growth inhibiting effects by targeting the EGFR pathway. In our present study, we observed that Met-F-AEA in combination with URB597 inhibits stress fiber and focal adhesion formations. Focal adhesions have been shown to connect the ECM to actin stress fibers thus re-organizing the matrix and regulating cell migration [36]. They are the main subcellular macromolecules that form close contacts between cells and the ECM. They play an important role in cell growth and migration. Transformation of epithelial cells into invasive carcinomas also depends on reorganization of the actin cytoskeleton that leads to stress fiber assembly [46]. Furthermore, the Met-F-AEA in combination with URB597 reduces EGF induced invasion and also downregulates MMP2 secretion, which mediates the invasiveness of cancer cells by aiding them to degrade the ECM and metastasize [38].

Our results show that Met-F-AEA in combination with URB597 causes G0/G1 cell cycle arrest mediated apoptosis, which is shown by reduction in G1/S phase checkpoint markers Cyclin D1 and CDK4 and apoptotic markers caspase-9 and PARP. This is important because in cancer, there exists an imbalance between cell proliferation and apoptosis which leads to tumor progression. Also, uncontrolled cellular growth due to aberrant EGFR signaling leads to dysregulation of the cell cycle, which involves G1/S checkpoint markers that are responsible for cell cycle progression [26, 47]. Furthermore, apoptosis, a programmed cell death mechanism [48], is usually associated with cell cycle arrest. Hence, our results suggest the ability of Met-F-AEA in combination with URB597 as an apoptosis inducing agent, controlling the cell survival/death cycle.

Overall, the results of our study suggest that the activity of the endocannabinoid anandamide increases when FAAH is inhibited, leading to enhanced anti-proliferative, anti-migratory and anti-invasive effects. We have also shown that Met-F-AEA in combination with URB597 crosstalks with EGF receptor to inhibit its activation, subsequently leading to downregulation of its signaling targets. These implicate that Met-F-AEA along with URB597 can be used as an effective therapeutic strategy for the treatment of EGFR overexpressing NSCLC. This is especially imperative considering the resistance of NSCLC to various chemotherapeutic drugs and its poor prognosis.

## MATERIALS AND METHODS

### Reagents and antibodies

Met-F-AEA and URB597 were purchased from Sigma Aldrich. Antibodies used were P-AKT, caspase-9, PARP, CDK4 (Cell Signaling), P-ERK, ERK, AKT, GAPDH, P-EGFR, EGFR (Santa Cruz), cyclin D1, Ki67 (Neomarkers) and FAAH (Cayman Chemicals).

### Cell culture

Human NSCLC cell lines- A459, A549, CALU1, H1299 and H460 were obtained from ATCC (American Type Culture Collection) and cultured in DMEM or RPMI-1640 (Corning Cellgro), supplemented with 10% heat inactivated fetal bovine serum (FBS), 5 units/mL penicillin, and 5 mg/mL streptomycin (Corning Cellgro). Cells were maintained at 37°C in a humidified 5% CO_2_ atmosphere incubator.

### Cell proliferation assay

Cells were seeded at a density of 5000 cells per well in 96 well plates and allowed to grow for 24h. Briefly, cells were treated with Met-F-AEA (10μM), URB597 (0.2μM) or in combination. Cell viability was measured using the MTT assay (Roche) as described in the supplier's protocols, based on the absorbance reading at 570nm with respect to the control.

### Clonogenic assay

Cells were seeded at low density in complete media (1000 cells per well in six well plates) and treated with vehicle, Met-F-AEA/URB597 or in combination for six days. After the treatment period, cells were washed with PBS and fixed with 4% formaldehyde for 20min, washed again, stained with 0.1% crystal violet and individual clones were manually counted under the microscope.

### Chemotaxis and wound healing assays

For the migration assay, 8μm transwell plates (Corning-Costar) were used. Briefly, cells were seeded in the upper chamber and chemoattractant EGF (100ng/ml) was added to the lower chambers as previously described. 12 hours after EGF stimulation, cells that migrated to the lower chamber were fixed, stained using Hema stain and counted. For the invasion assay, pre-coated Matrigel invasion chambers (BD Falcon) were used. After 24 hours of stimulation with EGF similar to that in migration assay, invaded cells were stained and counted. Wound scratch assay was done as described previously [49].

### FAAH small interfering RNA

H460 cells were transfected with FAAH siRNA (Dharmacon) using Lipofectamine, as per the manufacturer's recommendations. Scrambled non targeting siRNA was used as control. 36h after transfection, cells were treated with either Met-F-AEA or vehicle and subjected to MTT and western blot analysis.

### Immunofluorescence

Cells were seeded in 8 well chamber slides, treated, fixed and incubated with primary antibodies phalloidin or vinculin overnight at 4°C. After washing, cells were stained with Alexa Fluor- 488 or 594 conjugated secondary IgG antibodies and visualized under Olympus FV1000 Filter confocal microscope.

### Gelatin zymography

Gelatin zymography was used to determine MMP activity. Briefly, supernatants of treated cells were collected, concentrated using centrifugal filter units (Millipore) and run on Novex zymogram gel. The gel was then renatured and developed to visualize the bands as per the manufacturer's protocol (Life Technologies).

### Luciferase reporter assay

NF-kB activity was determined using NF-kB luciferase reporter assay (Promega). To determine the luciferase reporter activity, NF-kB luciferase constructs containing either the wild type or NF-kB vector were transfected in the pre-treated cells using lipofectamine. For internal control, cells were co-transfected with Renilla luciferase vector. 24h after transfection, EGF (100ng/ml) was added and then incubated for another 24h. Then, the cells were lysed to perform the luciferase assay as per the manufacturer's protocol.

### Western blotting

Cells were washed, lysed and protein estimation was performed using Bradford assay. Aliquots of cellular lysates (50μg) were electorphoresed on a 4-12% Novex SDS-PAGE, transferred to nitrocellulose membrane and blocked with 5% non-fat dry milk for an hour at room temperature. The membranes were then probed overnight with specific primary antibody (1:1000) overnight at 4°C. After washing thrice with 1X TBST, blots were exposed to secondary antibody (anti-mouse or anti-rabbit IgG-HRP, 1:2000) for an hour, washed thrice and detected using ECL chemiluminescence detection system (Thermo Scientific).

### Cell cycle analysis

Cells were trypsinized, washed with 1X PBS and fixed with 70% ethanol overnight at 4°C. Then, the cells were spun down, washed twice and incubated with 20μg/ml propidium iodide and 10 μg/ml RNAse for 30 min, washed and the DNA content was analyzed by flow cytometry.

### Apoptosis assay

Apoptosis was detected in cells using Click-iT TUNEL assay kit (Life Technologies). Cells were washed, fixed and treated with TdT enzyme for 1h. Then, the cells were stained with AlexaFluor 594 dye-labeled reaction buffer for 30min and detected under fluorescent microscope (Olympus).

### Mouse xenograft model

H460 cells (2×10^6^) in 100μl PBS were injected subcutaneously into the left flank of each male nude mouse. Once the tumors reached palpable size, they were treated with Met-F-AEA (5mg/kg), URB597 (1mg/kg) or in combination for 3 weeks. Tumor volume was calculated using the formula vol. = length*(width)^2^/2.

### Real Time PCR

RNA was isolated from tumors using TriZol reagent (Invitrogen). RT-PCR was performed as described earlier [50].

### Tissue Microarray (TMA) and Immunochemical (IHC) analyses

TMA and IHC were performed as described earlier [10, 50].

### Statistical analysis

Results were represented as mean ± SD which were analyzed using Student's two-tailed t test. A value of P<0.05 was considered to be statistically significant.

## SUPPLEMENTARY FIGURE


