# Supplementary material for: FAAH inhibition enhances anandamide mediated anti-tumorigenic effects in non-small cell lung cancer by downregulating the EGF/EGFR pathway
Source: Oncotarget. 2014 Feb 21;5(9):2475–86. doi: 10.18632/oncotarget.1723 (PMC4058020; doi:10.18632/oncotarget.1723)
Supplement: Supplementary file 1 [file oncotarget-05-2475-s001.pdf]

FAAH inhibition enhances anandamide mediated anti-tumorigenic effects in non-small cell lung cancer by downregulating the EGF/EGFR pathway – Ravi et al

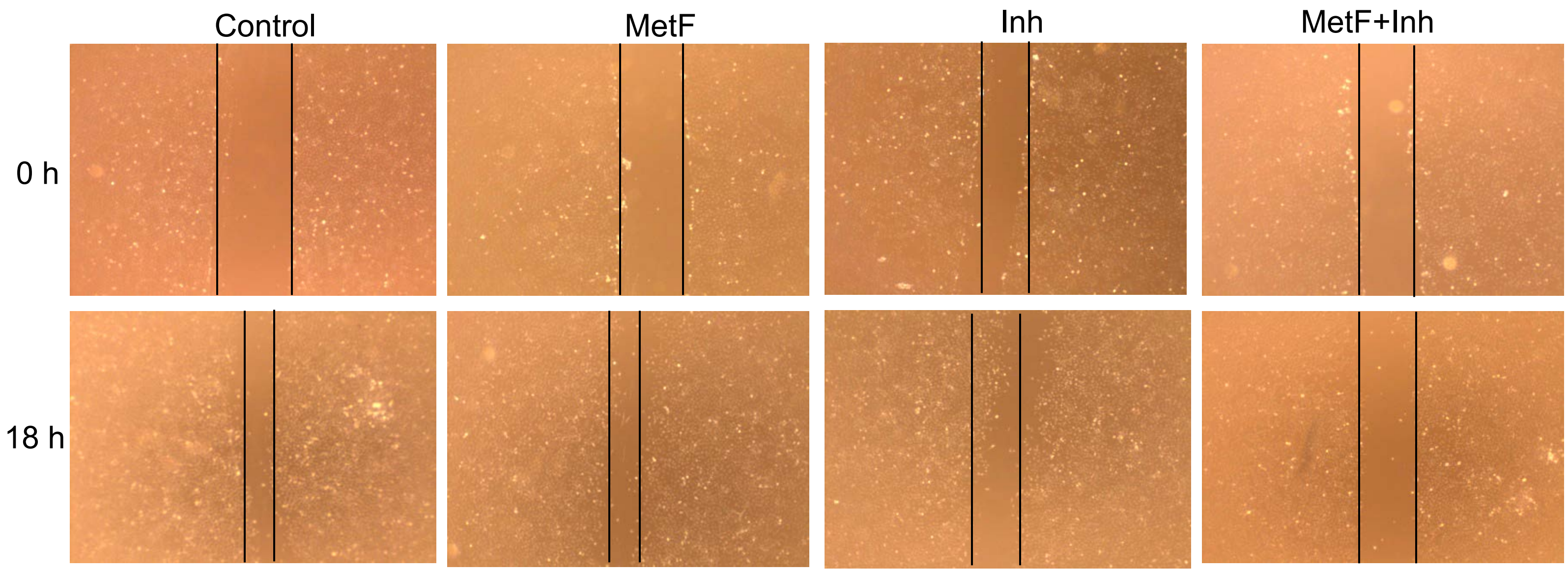

Fig S1: A549 cells were plated in six-well plates and pre-treated with control, Met-F-AEA (MetF, 10µM), URB597 (Inh, 0.2µM) or MetF+Inh for 24h. The monolayer was wounded by scoring a scratch with a sterile 200µl plastic tip, and then washed and fed with media containing EGF (100ng/ml)**.** After 18h, cells were photographed using a low magnification phase contrast microscope and the migratory ability was assessed qualitatively. Data represent the mean ± SD for each experiment repeated three times with similar results.
